# Type-I Interferon Responses: From Friend to Foe in the Battle against Chronic Viral Infection

**DOI:** 10.3389/fimmu.2016.00609

**Published:** 2016-12-19

**Authors:** Armstrong Murira, Alain Lamarre

**Affiliations:** ^1^Immunovirology Laboratory, Institut national de la recherche scientifique (INRS), INRS-Institut Armand-Frappier, Laval, QC, Canada

**Keywords:** type-I interferon, chronic viral infection, immunopathology, IFNAR, immunoregulation

## Abstract

Type I interferons (IFN-I) have long been heralded as key contributors to effective antiviral responses. More widely understood in the context of acute viral infection, the role of this pleiotropic cytokine has been characterized as triggering antiviral states in cells and potentiating adaptive immune responses. Upon induction in the innate immune response, IFN-I triggers the expression of interferon-stimulated genes (ISGs), which upregulate the effector function of immune cells (e.g., dendritic cells, B cells, and T cells) toward successful resolution of infections. However, emerging lines of evidence reveal that viral persistence in the course of chronic infections could be driven by deleterious immunomodulatory effects upon sustained IFN-I expression. In this setting, elevation of IFN-I and ISGs is directly correlated to viral persistence and elevated viral loads. It is important to note that the correlation among IFN-I expression, ISGs, and viral persistence may be a cause or effect of chronic infection and this is an important distinction to make toward establishing the dichotomous nature of IFN-I responses. The aim of this mini review is to (i) summarize the interaction between IFN-I and downstream effector responses and therefore (ii) delineate the function of this cytokine on positive and negative immunoregulation in chronic infection. This is a significant consideration given the current therapeutic administration of IFN-I in chronic viral infections whose therapeutic significance is projected to continue despite emergence of increasingly efficacious antiviral regimens. Furthermore, elucidation of the interplay between virus and the antiviral response in the context of IFN-I will elucidate avenues toward more effective therapeutic and prophylactic measures against chronic viral infections.

## Introduction

Upon viral infection, the immune response comprises a multi-layered coordination of effector functions broadly characterized as a progression from innate to adaptive immunity. Within the immunological milieu, Type I interferons (IFN-I) play a central role in driving an antiviral state in non-immune cells as well as orchestrating antiviral immune responses through: (i) inhibiting viral replication in infected cells in the innate stage of the immune response; (ii) activating and enhancing antigen presentation in the “early induced” immune response, and (iii) triggering the adaptive immune response through direct and indirect action on T and B cells that make up the memory response [reviewed in Ref. (1)]. Therefore, this cytokine acts as a master regulator whose induction in the early stages of viral infection modulates downstream signaling cascades that promote both pro-inflammatory and anti-inflammatory responses depending on the context of activation as discussed below. Whereas the protective role of IFNs has been widely characterized, emerging lines of evidence illustrate a deleterious effect borne by IFN-associated immunopathology (2, 3). These characterizations bear particular importance given the historic use and ongoing studies on IFN therapy in the treatment of chronic viral infections [e.g., HCV (4) and HIV (5–8)], autoimmune diseases [e.g., systemic lupus erythematosus (9)], and cancer (10–13). Whereas the advent of new therapies has spurred a trend toward IFN-free treatments in HCV, HIV, and oncology, IFN therapy is still considered to be a significant therapeutic agent due to its efficacy against HCV-associated complications [e.g., hepatocellular carcinoma (4)] and combinatorial effect in cancer therapy (14). In addition, cost restriction due to the price of emergent therapies also sustains the use of IFN-based therapies (15).

Described here in the context of viral infections, this review focuses on the course of IFN-I upon (i) elicitation; (ii) downstream signaling in various cell types, and (iii) the consequent binary effect on immunity. Collectively, we discuss the development of IFN-driven antiviral responses and key features that highlight potential targets toward effective treatment measures against chronic viral infections.

## Diversity in IFN-Associated Immune Responses

Type I interferons can be broadly characterized into three groups: IFN-I, Type II (IFN-II), and Type III (IFN-III) with subcategories therein based on gene loci of the IFN transcribing genes as well as difference in their cognate receptors. IFN-I is the largest and most well-characterized group with seven classes: IFNα, IFNβ, IFNδ, IFNϵ, IFNκ, IFNω, and IFNτ whereas IFN-II comprises IFNγ. IFN-I and IFN-II signal through IFNαR1/R2 (IFNAR) and IFNγR1/R2 (IFNGR), respectively. The last class IFN-III, otherwise classified as “IFN-like cytokines,” consists of interleukin (IL)-28A (IFNλ2), IL28B (IFNλ3), and IL29 (IFNλ1) and signals through IL-28RI/IL10R2 receptor chains [reviewed in Ref. (16)].

Upon pathogen-encounter, a plethora of cells are induced into IFN-I expression through recognition of pathogen-associated molecular patterns by putative pattern recognition receptors (PRRs), such as (i) toll-like receptors (TLRs) (17–24), (ii) retinoic-acid inducible gene I (RIG-I) (20, 25), (iii) melanoma differentiation-associated gene 5 (26), and (iv) nucleotide-binding oligomerization domain-containing protein (27). Consequent to PRR activation, signal transduction occurs through downstream transcription regulators called IFN regulatory factors (IRFs). This family of nine members, IRF1–IRF9 [see Table 1 in Ref. (28) for summary] offers yet another layer of diversity in the IFN response; convergence to and transcription by different sets of IRFs is determined by the nature of the sensing PRR, which resultantly determines the nature of the subsequent IFN responses.

The third layer of diversity entails the initiation of transcription by IRFs, which is facilitated by the variety of signal transduction pathways triggered upon elicitation of IFNs. Upon ligation of IFNAR, signal transducer and activator of transcription 1 (STAT1) and STAT2 are induced through phosphorylation by the tyrosine kinase 2 (TYK2) and Janus kinase (JAK1). Thereafter, STAT1 and STAT2 form a trimeric transcription factor, IFN-stimulated gene factor 3, by assembling with IRF9 that subsequently migrates into the nucleus to initiate transcription of IFN-stimulated genes (ISGs) by binding to the promoter regions known as IFN-stimulated response elements (ISRE) (29). Within this signal cascade lies combinatorial differences through which IFNs foster both proinflammatory and anti-inflammatory responses. For example, while signaling by IFN α/β through IFNAR typically leads to heterodimerization of STAT1 and STAT2, homodimerization between STAT1 and STAT3 may occur concurrently or alternatively upon IFNAR signaling. This different pairing of downstream STAT dimers therefore results in (i) the aforementioned engagement of ISRE toward antiviral responses (STAT1/3 heterodimers), (ii) the induction of pro-inflammatory responses by binding to IFNγ response elements (GAS) (STAT1 homodimers), or (iii) binding of STAT3-binding elements (SBE) to trigger an anti-inflammatory response (STAT3 homodimers) [reviewed in Ref. (30)].

Importantly, whereas STAT1 drives a pro-inflammatory, pro-apoptotic response, STAT3 dimerization favors an anti-inflammatory response that negatively regulates the action of STAT1 (31); we surmise that this is likely a homeostatic mechanism to counter the immunopathological effects of sustained IFN-associated pro-inflammatory responses. However, in the context of IL-6 cytokine signaling, the anti-inflammatory effect of STAT3 upon IFNAR signaling can also be counteracted through a negative feedback loop as well; this further underscores the multiplicity of interactions that govern IFN-I-associated signaling and its downstream effects (31). Lastly, in addition to the plethora of molecular interactions, the presence of IFN-I receptors on various cell types [e.g., hematopoietic stem cells (32, 33), macrophages (34–36), dendritic cells (DCs) (37–43), and natural killer (NK) cells (35, 44–47)] further enhances the impact of IFN-I upon induction.

## IFN-I Responses in Chronic Infection

It is important to consider that the antiviral effects of IFN-I have been primarily made in the framework of an acute infection in which the intricate interplay of well-timed and tightly regulated IFN responses functions optimally toward resolution of an infection. What are the effects of prolonged IFN-I production such as in the case of chronic infections? This is an open question that is gaining increasing traction based on emerging data on the deleterious effects of IFN-I in the chronic setting. Importantly, various combinations of IFN-I are used as therapeutic measures particularly in chronic infections. Given the historical and continued use in clinical applications, this is a crucial factor to consider given the multifaceted ways in which IFN elicitation and response are regulated in a fine balance whose perturbation bears impact ranging from hematopoiesis to mature differentiated adaptive immune responses.

## IFN-I Responses in Lymphocytic Choriomeningitis Virus (LCMV) Infection

The deleterious effect of IFN-I responses has been brought into sharper focus more recently by two independent studies using a chronic (LCMV-Clone 13) versus acute (LCMV-Armstrong) infection model, which revealed that viral persistence was diminished by *in vivo* IFNAR blockade (2, 3). In their analyses, Teijaro et al. illustrated that IFNAR blockade led to the rescue of IFNγ^+^ CD4 T cells, which as discussed comprise the T helper 1 (T_H_1) cellular subsets that potentiate cytotoxic T lymphocyte (CTL) responses. Strikingly, this study revealed that the size of the CTL subpopulation was not changed despite the enhanced viral clearance observed; thus, functional quiescence (similar to exhaustion) in the face of sustained IFN-I signaling partially facilitates impairment of viral clearance by CTLs. A significant finding in these studies was that in addition to the net detrimental effects of sustained IFN-I, elicitation of high concentration of the cytokine early in the course of infection correlated with viral persistence.

As outlined, IFN-related mechanisms are governed by feedback loops to ascertain homeostasis and prevent immunopathology. An example of these coordinate measures is observed in the switch from T_H_1 responses toward T follicular helper (T_FH_) cells. Fahey et al. originally depicted this transition using LCMV. By comparing LCMV-Armstrong versus LCMV-Clone 13, they observed that while mice infected with an acute strain of the virus did not bear any aberrant elevation of T_FH_ markers, the chronic phase of LCMV-Clone 13 infection exhibited increased proportions of T_FH_ cells depicted by putative markers such as (i) CXCR5; a B cell homing chemokine receptor; (ii) ICOS; an inducible T cell costimulatory molecule; and (iii) inducible T cell costimulatory OX40, also known as TNFRSF4. A significant distinction to make here is that T_FH_ cells were also present in the acute infection but these abated upon resolution of the infection (48). In follow-up analyses, Osokine and colleagues revealed that this switch occurred in an IFN-I-dependent manner wherein the absence of IFN signaling, T_H_1 responses were maintained; in the presence of IFN-I, the cytokine actively suppressed the emergence of *de novo* T_H_1 cells in a pre-programed function that occurred early in the priming stages of the infection (49). The underlying principle behind this transition is to curb the T_H_1 response, which triggers IFNγ expression that in turn activates CTLs and NK cells. From a homeostatic point of view, prolonged effector function of these cells may lead to excessive cytotoxicity and other detrimental effects resulting in host tissue damage.

However, in the event of viral persistence, this skew toward T_FH_ responses results in a number of aberrant responses that hinder viral clearance. Decades-long characterization of CTL exhaustion has been at the forefront of chronic-infection immune response perturbations [(50), reviewed in Ref. (51)]. Initially characterized in LCMV infection as well, exhausted CTLs were observed to be refractory to activation signals, prone to apoptosis, and feature an upregulation of inhibitory markers (52–56). Notably, the aforementioned switch to T_FH_ from T_H_1 results in diminished activation of CTLs based on the resultant reduction of the second activation signal required to fully activate naïve CTLs. As shown by Fuller et al., the absence of T_H_1 licensing (57) along with the reduction of IFNγ due to contraction of T_H_1 cell populations as infection progresses toward chronicity leaves CTLs in a pseudoactivated state characterized as exhaustion.

That the T_FH_ subpopulation is atypically expanded in chronic infections (48, 49) also imposes dysregulation on their close immunological counterparts, the B cells. In the context of a chronic infection, perturbations such as atypical B-cell subpopulations, hypergammaglobulinemia (HGG), and polyspecificity are well characterized (58–66). Along with others, we observed the extensive impact of IFN-mediated responses on humoral immunity both directly and indirectly in the context of viral persistence. In our study, we found that in addition to the indirect T_FH_-associated humoral response perturbation, there was a direct IFN-I-mediated effect on B cells (67). Comparing LCMV-Clone 13 versus LCMV-WE (acute), we observed sustained ablation of antigen specificity against a secondary immunogen, nitrophenylacetyl-chicken gamma globulin (NP-CGG), in the former whereas the latter only showed transient impact on antigen specificity. Furthermore, we also evaluated antigen specificity of NP-CGG in the context of vesicular stomatitis virus (an acute infection), which remained unchanged. Remarkably, we observed the rescue of antigen specificity upon IFNAR blockade in addition to a recovery of lymphoid architecture similar to previous studies (2, 3, 67, 68). Most importantly, we also assessed the humoral response using a chimeric mouse model comprising reconstitution of irradiated B6 mice with a mix of bone marrow cells from J_H_T (B-cell deficient) (69) and IFNAR^−/−^ mice. Here, we observed that in the absence of IFNAR signaling in B cells, neutralizing antibodies (nAbs) against LCMV were elicited more robustly and earlier than in wildtype mice and control J_H_T/B6 chimeras. These results are in agreement with previous findings by Price et al. who also showed that in the absence of IFN-I signaling, nAb responses against influenza virus developed more efficiently (70). Recently, the direct effect of IFN signaling on B cells has also been illustrated using *Leishmania donovani*, which is the etiological agent of the chronic disease, visceral leishmaniasis. In this study, Silva-Barrios et al. illustrated that B-cell activation occurred in an IFN-associated, TLR-dependent manner that culminated in disruption of the humoral immune response that typifies other chronic infections. Similar to our findings, they also observed the reduction of HGG upon B-cell-specific IFNAR knockout in mice (71), which further supports the role played by IFN signaling toward this phenomenon.

## IFN-I Responses in HCV Infection

In the perspective of human infection, the role of IFN responses is particularly important based on the widespread use of IFN therapy against chronic viral diseases such as HCV (4), HIV (5–8, 72), and more broadly in clinical setting such as systemic lupus erythematosus (9), melanoma, and other neoplastic indications [(11–13), reviewed in Ref. (10)]. It is important to state that the standard of care in HCV is slowly moving away from IFN-based therapy, whereas HIV anti-retroviral therapy is almost entirely IFN-free except in impoverished regions. Of note, although some of these conditions are non-viral infections, they all feature antigenic persistence and therefore resemble chronic viral infections despite different etiologies. Given the pervasive influence of IFN-I responses and data revealing both positive as well as negative effects of the cytokine, it is also imperative to critically delineate the effect of IFN-I in chronic disease settings.

Generally, the immunopathology associated with IFN-I, e.g., aberrant cellular populations, inadequate immune responses, and disrupted cytokine environments are also observed in HCV. On a molecular level, most characterizations of IFN cellular responses have been made using *in vitro* models, e.g., HCV pseudoparticles (73) and HCV cell culture (74, 75) systems whereby the impact of IFN is observed in the context of both endogenous expression in cell culture and exogenous supplementation akin to administration of therapy. Detection of viral RNA occurs through typical PRR-recognition pathways [(76, 77), reviewed in Ref. (78)], upon which upregulation of ISGs occurs (79). Interestingly, researchers observed a coincidence between low response rates to IFN treatment in patients with high baseline levels of IFN in their plasma (80). In this study, Sarasin-Filipowicz and colleagues revealed that hepatocytes obtained from chronically infected, non-responder patients bore non-responsive signaling to IFN treatment *ex vivo*. Similarly, evidence of attenuation in IFN responses in the chronic phase of HCV is also suggested by the prevalence of ineffective CTL responses upon delayed induction of IFNα-therapy, whereas functional effector activity was maintained or restored in spontaneous resolvers or responders, respectively (81). At the transcriptional level, clues toward IFN-resistance are posited by the discovery of proviral ISGs whereby recent work has shown that some ISGs work to promote the HCV resistance in cell culture. For example, overexpression of ubiquitin-specific protease 18 (USP-18), which functions as a negative regulator of IFN signaling drives, a proviral response highlighted by evidence of up regulation in HCV patients who do not respond to IFN treatment (82). Conversely, USP18^−/−^ mice are resistant to viral infection (83). Here, USP18 works in concert with ISG-15, therefore inhibiting effective JAK/STAT signaling; based on the significance of this signaling pathway toward effective IFN signaling, the expression of these ISGs results in diminished IFN responses and counterintuitively facilitate HCV replication (84, 85). Important to note here is that transcription of both antiviral and proviral ISGs are driven by ligation of IFN receptors. Similarly, the presence of “negative regulators” such as these is therefore likely a negative feedback mechanism, which when functioning optimally reverts the host immunological milieu to “steady-state”. However, against chronic infection, the presence of such processes also contributes to desensitization to therapeutic IFN-administration in HCV patients with high levels of IFN expression (86). In this setting, the consequent evocation of ISGs such as USP-18 and ISG-15 renders the patients non-responsive to therapy (87, 88). This feature also underscores the possibility that efficacious virologic responses against persistent infection are blunted over time due to the presence of proviral ISGs. Along with the IFN-led dysregulation described in the LCMV model, the presence of dysregulation at the ISG level further renders the immune response in a state of flux and incapable of clearing the infection.

## IFN Responses in HIV Infection

The progression of the HIV-associated IFN-I response closely mirrors that observed upon HCV infection. This evolution has been elegantly laid out using a simian immunodeficiency virus (SIV) model in rhesus macaques. In this study, Sandler et al. observed that IFN blockade *in vivo* accelerated advancement to AIDS with unchecked SIV replication whereas IFNα administration conferred resistance to the host upon challenge (89). However, in line with the observation of desensitization discussed in HCV, they also observed that sustained IFN administration led to a reversal of host resistance to infection and conversely, resembled the IFN blockade scenario in which the SIV reservoir was enlarged along with CD4^+^ T-cell depletion and AIDS. Notably, CD4^+^ T-cell depletion in this setting could be a function of the cellular tropism of the virus rather than solely the direct effect of IFN-mediated effects.

Furthermore, a wealth of research has also underscored the elevated IFN signature observed in the chronic stage of HIV infection, which correlates with high levels of viral load and thus, failed viremia control. Following transcriptome analyses on CD4^+^ T cells, Rotger et al. found that ISGs were upregulated in untreated patients relative to patients on therapy and healthy controls. In addition, upon induction of antiretroviral therapy and reduction of viremia, the ISG profiles in patient T cells reverted to those observed in the cohorts of HIV-infected individuals who maintain a CD4 T cell count of ≥500 (elite controllers) whose IFN level, and resultantly ISG expression is at a lower baseline (90). These findings were supported by previous findings of ISG upregulation *in vitro* and *in vivo* in CD4^+^ T cells from chronically infected HIV^+^ patients relative to healthy controls (91). Furthermore, despite similarity in expression levels in the acute phase of infection, the absence of hyperactivated IFN expression is a distinctive factor between pathogenic and non-pathogenic forms of SIV; while pathogenic SIV_mac_ in rhesus macaques features an elevated IFN signature and resultant disease and the non-pathogenic SIV_agm_ and SIV_smm_ in African green monkeys and Sootey mangabeys, respectively, neither exhibit aberrant IFN upregulation nor immune activation (92–94).

Lastly, the differences between pathogenic and non-pathogenic forms of SIV are partially driven by distinct signaling potentials through PRRs in pDCs (94); strong signaling through TLRs is observed in pathogenic SIV, which results in a surge of IFN that further propagates an immunopathogenic response as outlined in the various scenarios described above.

## Closing Remarks and Outlook

**Figure 1:**
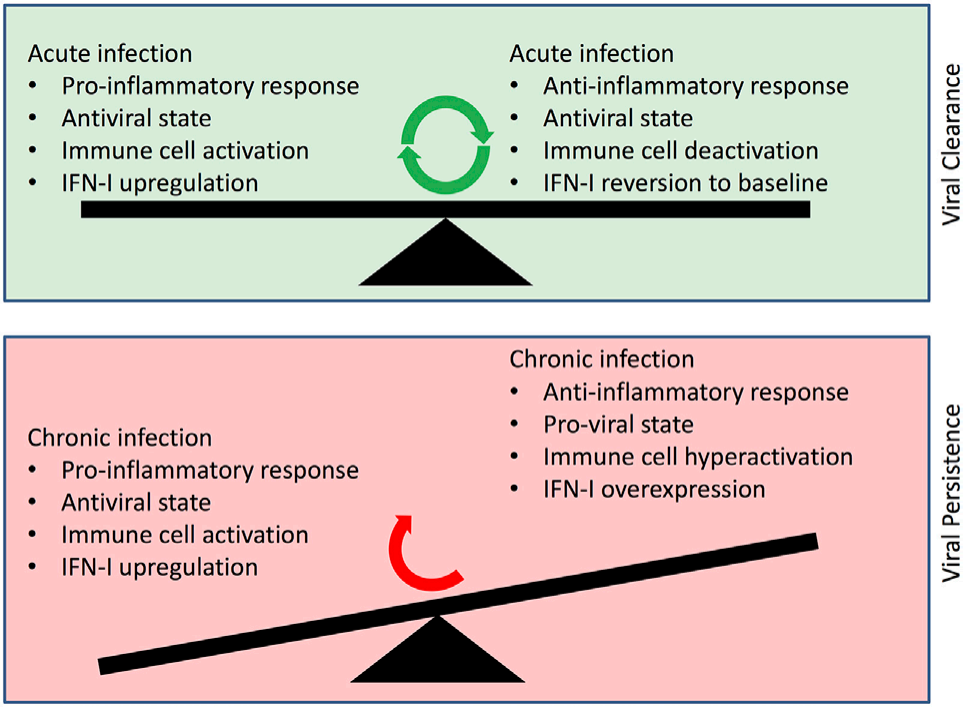
**Depiction of changes in Type I interferon (IFN-I) roles in viral clearance vs. viral persistence**. (Upper panel) Balance of functional IFN-I signaling, which results in viral clearance within the context of an acute infection. (Lower panel) Dysfunctional IFN-I signaling in the face of a chronic infection resulting in aberrant immune cell activation and viral persistence.

It is important to note that causality between prolonged IFN expression and viral persistence is yet to be fully determined: does prolonged IFN diminish the immune response leading to viral persistence or does persistent infection lead to prolonged IFN expression whose dysregulation of immune responses is misconstrued as cause rather than effect? Nevertheless, the dizzying network of IFN-activating and IFN-inhibiting responses highlights the complexity in elucidating the exact nature of the IFN-related immunopathology in chronic infection (summarized in Figure 1). Intuitively, disruption of the delicate balance using exogenous IFN may result in less efficacious responses and adverse event profiles in therapeutic administration of IFN (95, 96). On the contrary, the multiplicity of pathways and molecules offers avenues that can be useful toward more effective therapeutic approaches by specific targeting of the deleterious moieties. For example, targeting proviral ISGs may offer an incisive approach toward triggering effective IFN responses and through their rescue, obviate exogenous IFN administration. From a prophylactic perspective, induction of nAbs in the absence of IFN signaling in B cells offers insight into the mechanisms that drive the delayed effective humoral response in diseases such as HIV and HCV. Given that the emergence of broadly nAbs against these chronic infections is delayed and in a highly altered immunological milieu, delineating the role of IFN-I facilitates a more comprehensive understanding of the conditions present during elicitation of broadly nAbs. In this regard, it is tempting to speculate that perhaps modulation of the IFN response along with the appropriate immunogen may advance vaccine work in these chronic infections along with other prophylactic measures as well. Altogether, these emergent insights bear significant impact on our understanding of the role of IFN-I in the immune response and importantly, its use in therapeutic settings. Guided by these findings, future work will more clearly determine the delicate balance that tips IFN responses from friend to foe.

## Author Contributions

AM contributed to the conceptualization of the subject, literature search, and writing the manuscript. AL contributed to the conceptualization of the subject, critical review of compiled literature, and writing the manuscript.

## Conflict of Interest Statement

The authors declare that the research was conducted in the absence of any commercial or financial relationships that could be construed as a potential conflict of interest. The reviewers MR and NF and handling Editor declared their shared affiliation, and the handling Editor states that the process nevertheless met the standards of a fair and objective review.
